# Modified Zexie decoction improves phlegm-dampness type stage I hypertension by regulating the gut-immune-kidney axis

**DOI:** 10.3389/fphar.2025.1578815

**Published:** 2025-06-18

**Authors:** Zeqi Hu, Jiujie Jia, Yiyan Su, Yihao Gu, Bingbing Cheng, Ninghua Jiang

**Affiliations:** ^1^ College of Pharmaceutical Science, Zhejiang University of Technology, Hangzhou, Zhejiang, China; ^2^ College of Pharmaceutical Science, Zhejiang Chinese Medical University, Hangzhou, Zhejiang, China; ^3^ Department of Pharmacy, The Second Affiliated Hospital of Jiaxing University, Jiaxing, Zhejiang, China

**Keywords:** MZXD, immune, phlegm-dampness type stage I hypertension, gut-immune-kidney axis, gut microbiota, renal function

## Abstract

**Indroduction:**

Hypertension pathogenesis increasingly implicates gut microbiota‐host crosstalk, where immune regulation bridges gut dysbiosis and renal dysfunction. Phlegm-dampness hypertension—a prevalent Traditional Chinese Medicine (TCM) syndrome typified by metabolic, immune, and renal anomalies—demands novel interventions. Modified Zexie Decoction (MZXD), a TCM formula with documented antihypertensive and anti-inflammatory properties, may target the gut‐immune‐kidney axis. This study investigated MZXD’s efficacy in Stage I phlegm-dampness hypertension via this axis.

**Methods:**

Thirty Stage I phlegm‐dampness hypertension patients received MZXD for 42 days. Pre‐ and post‐treatment measurements included blood pressure, serum immune markers, renal function parameters, gut microbiota, and short‐chain fatty acids (SCFAs).

**Results:**

MZXD significantly reduced systolic/diastolic blood pressure (P < 0.05). Gut microbiota diversity increased, shifting toward healthy profiles. SCFA levels rose markedly (P < 0.01). Immune markers (P < 0.05) and renal function improved (P < 0.05).

**Discussion:**

MZXD alleviates phlegm‐dampness hypertension by modulating the gut-immune‐kidney axis: enhancing microbial diversity, boosting SCFAs, attenuating inflammation, and improving renal function. This first systematic validation positions MZXD as a promising multi-target therapy. Future studies should explore clinical scalability and mechanistic depth.

## Graphical Abstract

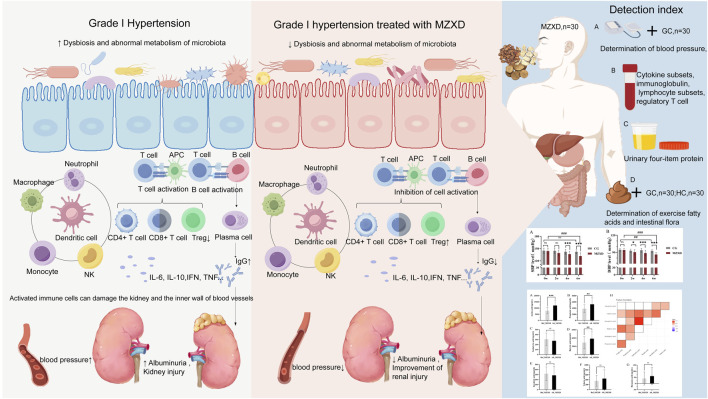

## 1 Introduction

Hypertension is the most prevalent risk factor for cardiovascular diseases (Carey et al., 2018). With the growth and aging of the global population, the prevention and management of hypertension have become significant public health issues worldwide. The majority of patients present with Stage I hypertension (Tamisier and Lévy, 2017), defined by the “2018 Chinese Hypertension Guidelines” as a systolic blood pressure of 140–159 mmHg and/or a diastolic blood pressure of 90–99 mmHg, representing an early stage of hypertension. Although its symptoms may not be apparent, without timely intervention, it can progress to more severe hypertension, thereby increasing the risk of cardiovascular events. Due to the impact of unhealthy lifestyles, phlegm-dampness type hypertension is a common syndrome type of hypertension (Zhang et al., 2021). Most patients exhibit various forms of metabolic abnormalities, immune disorders, and renal impairment. Given that it is a multifactorial clinical condition, its exact pathogenesis has not yet been elucidated. Many current treatments primarily focus on correcting elevated blood pressure as the main symptom, while neglecting the underlying risks of the disease.

Recent studies have identified immune cells as key mediators in hypertension (Rudemiller and Crowley, 2017). T cells, B cells, monocytes, and natural killer cells are all associated with hypertension; they accumulate in the three key interacting organs responsible for hypertension (the heart, kidneys, and blood vessels), inducing chronic inflammation, which disrupts the normal functions of these organs in regulating blood pressure (Drummond et al., 2019; Guzik et al., 2024). The gut harbors the largest proportion of immune cells in the body (Esterházy and Mucida, 2019). A reduction in the richness and diversity of gut microbiota leads to metabolic disturbances and an imbalance in gut homeostasis, altering gut immune status. Mature immune cells can be activated by gut microbiota in surrounding lymphoid organs and influence renal function through the bloodstream (Esterházy and Mucida, 2019; Guo, 2021; Tian and Liang, 2021), leading to decreased renal function, reduced glomerular filtration rate, increased proteinuria, and damage to the glomeruli and renal tubular interstitium (Yang et al., 2018). These pathological changes in the gut and kidneys largely contribute to the development of hypertension (Ghosh and Valdes, 2023; Hu et al., 2017).

Zexie Decoction (ZXD) is a classic formula from “Jin Gui Yao Lue”, composed of two herbs, Zexie (*Alisma plantago-aquatica* L.) and Baizhu (*Atractylodes macrocephala* Koidz.), with thousands of years of clinical application. Previous studies have extensively demonstrated that ZXD and its various modified formulas can improve conditions such as hyperlipidemia and non-alcoholic fatty liver disease through gut microbiota modulation (Yan-qion, 2015; Zhang et al., 2022; Zhang et al., 2024), but its application in hypertension remains understudied. Phlegm-dampness type Stage I hypertension is often characterized by internal obstruction of phlegm-dampness and phlegm-heat stagnation. According to TCM theory, Shanzha (*Crataegus pinnatifida* Bunge) promotes digestion, strengthens the stomach, regulates qi, and disperses blood stasis. Previous studies have shown that its extracts can improve blood pressure by modulating immune imbalances (Cheng et al., 2023) and have antihypertensive effects in patients with mild essential hypertension (Walker et al., 2002). Therefore, MZXD was developed by adding Shanzha to the original ZXD based on TCM theory and existing research. The combined effects of the three herbs aim to dry dampness, resolve phlegm, and strengthen the spleen and stomach, effectively improving phlegm-dampness syndrome. However, it remains unclear whether MZXD can improve phlegm-dampness type Stage I hypertension and its possible mechanisms. Since MZXD is administered orally, we hypothesize that the gut microbiota is the primary target of MZXD. Given the established link between gut microbiota-host crosstalk and hypertension, most studies have focused on their correlation, with a lack of in-depth exploration of specific immune mechanisms. Thus, we launched the first clinical study of the effect of MZXD on the gut-immune-renal axis of a specific hypertensive subtype, hypothesizing that MZXD, while benefiting gastrointestinal health, can reshape gut homeostasis by improving gut microbiota and promoting the production of SCFAs, thereby inhibiting immune activation, reducing inflammatory damage, and consequently minimizing renal impairment.

## 2 Materials and methods

### 2.1 General data

This study randomly selected 60 patients with phlegm-dampness type Stage I hypertension who visited the Second Affiliated Hospital of Jiaxing University between January 2023 and March 2024. These 60 patients were randomly divided into two groups: 30 patients received a healthy lifestyle intervention for 42 days, and another 30 patients were treated with MZXD for 42 days. Additionally, 30 healthy volunteers were recruited as a control group. To protect the participants, those not receiving medication were not given placebos or subjected to blood parameter testing; only fecal samples were collected after the healthy lifestyle intervention. The study was approved by the Ethics Committee of the Second Affiliated Hospital of Jiaxing University, Zhejiang, China (Ethics Approval Code: JXEY-2022XJ170), and all participants signed informed consent forms.

### 2.2 Inclusion criteria

Patients meeting the following criteria were included in the study:

1. Met the diagnostic criteria for primary Stage I hypertension and were confirmed as having the phlegm-dampness type by two certified TCM practitioners.
2. Aged between 30 and 60 years, regardless of gender.
3. Had not taken any medication (including herbal medicine, Chinese patent medicine, or Western medicine) within 2 weeks prior to treatment.
4. Did not participate in any other studies.
5. Both the patients and their family members were fully informed about the study and provided signed informed consent.

### 2.3 Exclusion criteria

Patients meeting any of the following conditions were excluded from the study:

1. Secondary hypertension.
2. Severe cardiac insufficiency.
3. Severe hepatic or renal insufficiency.
4. Pregnant or breastfeeding women.
5. History of cerebrovascular disease.
6. Immune-related diseases.
7. Abnormal blood biochemical tests or infectious diseases, such as HIV carriers or patients positive for hepatitis B surface antigen.
8. Recent history (within the past 2 weeks) of high fever, gastritis, diarrhea, inflammatory bowel disease, irritable bowel syndrome, or intestinal resection.
9. Inability to comply with the medication regimen or refusal to cooperate with the treatment.

### 2.4 Treatment method

The control group (CG, n = 30) and the healthy control group (HC, n = 30) received a 42-day healthy lifestyle intervention (Table 1). The Modified Zexie Decoction treatment group (Bef_MZXD represents the group before MZXD treatment; Aft_MZXD represents the group after MZXD treatment, n = 30) received MZXD in addition to the 42-day healthy lifestyle intervention. MZXD is formulated in the proportion outlined in Table 2 and administered orally before breakfast and after dinner (150 mL per dose). All herbs come from the Pharmacy Department of Jiaxing Second Hospital (Jiaxing, China) and are certified by Professor Jiang Ninghua (Zhejiang University of Traditional Chinese Medicine and Jiaxing Second Hospital). The specimen of the certificate (voucher number: MZXD-202307001) is deposited in the specimen room of the traditional Chinese medicine pharmacy of the Second Hospital of Jiaxing. MZXD is uniformly prepared by the Traditional Chinese Medicine Preparation Department of Jiaxing Second Hospital, and its decoction process and quality control are in line with the “Hospital Traditional Chinese Medicine Decoction Pieces Management Specifications”.

**TABLE 1:** Healthy lifestyle intervention.

Intervention	Specific measures
Sodium reduction	Daily salt intake ≤6 g (≈1 standard beer bottle cap) Monitor hidden salt sources (pickled foods, monosodium glutamate, soy sauce, etc.)
Balanced diet	Emphasize fruits, vegetables, low-fat dairy, whole grains, and plant-based proteins Reduce saturated fats and trans fatty acids intake
Weight control	Target BMI <24 kg/m^2^ Waist circumference <90 cm (men)/<85 cm (women)
Regular exercise	Moderate-intensity exercise 30 min per session, 5–7 sessions weekly
Smoking cessation	Advise complete smoking cessation Avoid secondhand smoke exposure
Alcohol restriction	Recommend complete alcohol abstinence Current drinkers should be advised to abstain
Stress management	Implement stress reduction techniques Maintain psychological wellbeing

**TABLE 2:** The composition of MZXD.

Chinese name	Latin name	Genus family	Batch number	Part used	Proportion (g)
Zexie	*Alisma plantago-aquatica* L.	Alismatidae	1230716Y	Rhizome	15
Baizhu	*Atractylodes macrocephala* Koidz.	Asteraceae	2230904X	Rhizome	6
Shanzha	*Crataegus pinnatifida* Bunge	Rosaceae	22203024Y	fruit	9

Note: The plant name has been checked with http://mpns.kew.org.

The chemical composition of traditional Chinese medicine compound prescription is complex. In order to clarify the material basis of MZXD, the chemical constituents of MZXD were identified by ultra high performance liquid chromatography-tandem mass spectrometry (UPLC-MS/MS). The detailed analysis results can be found in the supplementary document.

### 2.5 Blood pressure measurement

Changes in office blood pressure, including systolic and diastolic blood pressure, were observed in both the MZXD treatment group and the CG before and after the intervention. Blood pressure was measured on the right brachial artery of the patient’s upper arm at the same time in the early morning before and after treatment. Patients rested quietly for 10 min before blood pressure measurement, and the blood pressure was measured three times consecutively. The average of the three readings was taken as the final value.

### 2.6 Determination of blood parameters and urine

In the MZXD treatment group, fasting venous blood (2–5 mL) was collected from the patients before and after treatment using vacuum tubes containing coagulant and separation gel (yellow cap tubes), typically from the antecubital vein. The analysis included the detection of cytokine subsets, immunoglobulins, lymphocyte subsets, regulatory T-cell levels, and renal function. The immune turbidimetric method was used for testing. After fasting for 10 h, the first-morning urine sample was collected and stored at 4°C, and testing was completed within 2 h.

### 2.7 Fecal collection and DNA extraction

Fresh fecal samples (approximately 3 g) were collected from the CG, HC, Bef_MZXD, and Aft_MZXD. The samples were immediately placed into sterile cryogenic tubes, rapidly frozen in liquid nitrogen, and stored in a −80°C freezer. DNA from the fecal microbiota was extracted using the MagBeads FastDNA^®^ Kit for Soil (Catalog No.: 116564384, MP Biomedicals, United States). Agarose gel electrophoresis was used to assess the molecular size of the extracted DNA, and quantification was performed using a Nanodrop spectrophotometer. The extracted DNA was then used for microbial diversity sequencing and sequence processing.

### 2.8 16S rRNA high throughput sequencing analysis

High-throughput sequencing of the V3-V4 hypervariable regions of the 16S rRNA gene was performed using the Illumina platform. The standard bacterial 16S V3V4 primers, ACT​CCT​ACG​GGA​GGC​AGC​A and GGACTACHVGGGTWTCTAAT, were used to amplify the 16S rRNA gene through polymerase chain reaction (PCR). Before analysis, sequences were demultiplexed and quality-filtered using the QIIME2 platform, resulting in Illumina reads with an average length of 500 bp. Sequences were analyzed using QIIME2 (v2020.2), and chimeric reads were checked. Sequences with 100% similarity were grouped into amplicon sequence variants (ASVs).

### 2.9 Determination of SCFAs

Targeted metabolomics analysis using gas chromatography-mass spectrometry (GC-MS) (Agilent 7890B-5977MSD, Agilent, United States) was employed to collect data and explore fecal metabolic changes in patients through multivariate statistical analysis. Approximately 0.5 mg of the lyophilized sample was taken, and the target indicators were extracted using ultrapure water with thorough ultrasonic extraction. All samples were processed according to the standard instructions and requirements of GC-MS. The concentration of SCFAs was identified by comparing the results with the NIST spectral library and SCFAs standards using the workstation data processing system. Peak extraction from the raw data was performed using Quant Analysis software, and the peak areas of each compound were obtained. The content of SCFAs (acetate, propionate, butyrate, isobutyrate, valerate, isovalerate, isocaproate, caproate) in fecal samples was calculated based on the standard curves.

### 2.10 Statistical analysis

Statistical analysis was performed using SPSS Statistics 27 (IBM Software, New York, United States), and graphs were generated using GraphPad Prism 9 (GraphPad Software, San Diego, California, United States). Based on the results of normality tests, paired t-tests and one-way analysis of variance (ANOVA) were used for normally distributed data. For data that did not follow a normal distribution, the Wilcoxon rank-sum test and the Kruskal–Wallis test were employed. Additionally, categorical variables were analyzed using the Chi-Squared Test, *p* < 0.05 was considered statistically significant.

## 3 Results

### 3.1 Baseline characteristics

The 60 patients with phlegm-dampness type Stage I hypertension were randomly divided into two groups: 30 patients received a 42-day healthy lifestyle intervention, and another 30 patients were treated with MZXD for 42 days. Additionally, 30 healthy volunteers were recruited. The baseline characteristics of the participants are shown in Table 3, including gender, age, body mass index (BMI), smoking status, drinking status, and disease duration. The mean ages of the three groups were 47.07 ± 8.21 years, 49.37 ± 6.12 years, and 45.13 ± 8.62 years, respectively. The mean BMIs of the three groups were 24.91 ± 3.54 kg/m^2^, 24.24 ± 2.58 kg/m^2^, and 22.73 ± 2.52 kg/m^2^, respectively. Apart from the metabolic disturbances observed in patients with phlegm-dampness type Stage I hypertension, their BMI was slightly higher than that of the healthy volunteers. At baseline, there were no significant differences among the three groups in terms of gender, age, smoking status, drinking status, and disease duration (*p* > 0.05).

**TABLE 3:** Baseline clinical characteristics of the study population.

Variable	CG (n = 30)	MZXD (n = 30)	HC(n = 30)	*x* ^*2*^ */z/F*	*p-*value
Sex, n (%)				4.267	0.118
Man	15 (50.0%)	19 (63.3%)	11 (36.7%)		
Female	15 (50.0%)	11 (36.7%)	19 (63.3%)		
Age (years), mean ± SD	47.07 ± 8.21	49.37 ± 6.12	45.13 ± 8.62	3.466	0.117
BMI (Kg/m^2^), mean ± SD	24.91 ± 3.54	24.24 ± 2.58	22.73 ± 2.52	4.399	0.015*
Smoking, n (%)				1.555	0.459
Yes	3 (10.0%)	5 (16.7%)	2 (6.7%)		
No	27 (90.0%)	25 (83.3%)	28 (93.3%)		
Alcohol consumption >40 g/day, n (%)				1.417	0.492
Yes	3 (10.0%)	3 (10.0%)	1 (3.3%)		
No	27 (90.0%)	27 (90.0%)	29 (96.7%)		
Duration of illness (months)				0.113	0.945
≤1	22 (73.3%)	23 (76.7%)	N/A		
>1,≤2	6 (20.0%)	5 (16.7%)	N/A		
>2,≤3	2 (6.7%)	2 (6.7%)	N/A		

Note: Comparison between groups, *p < 0.05. CG: control group; MZXD: treatment group with modified Zexie decoction; HC: healthy control group.

### 3.2 Effects of MZXD on blood pressure in patients with phlegm-dampness type stage I hypertension

**FIGURE 1:**
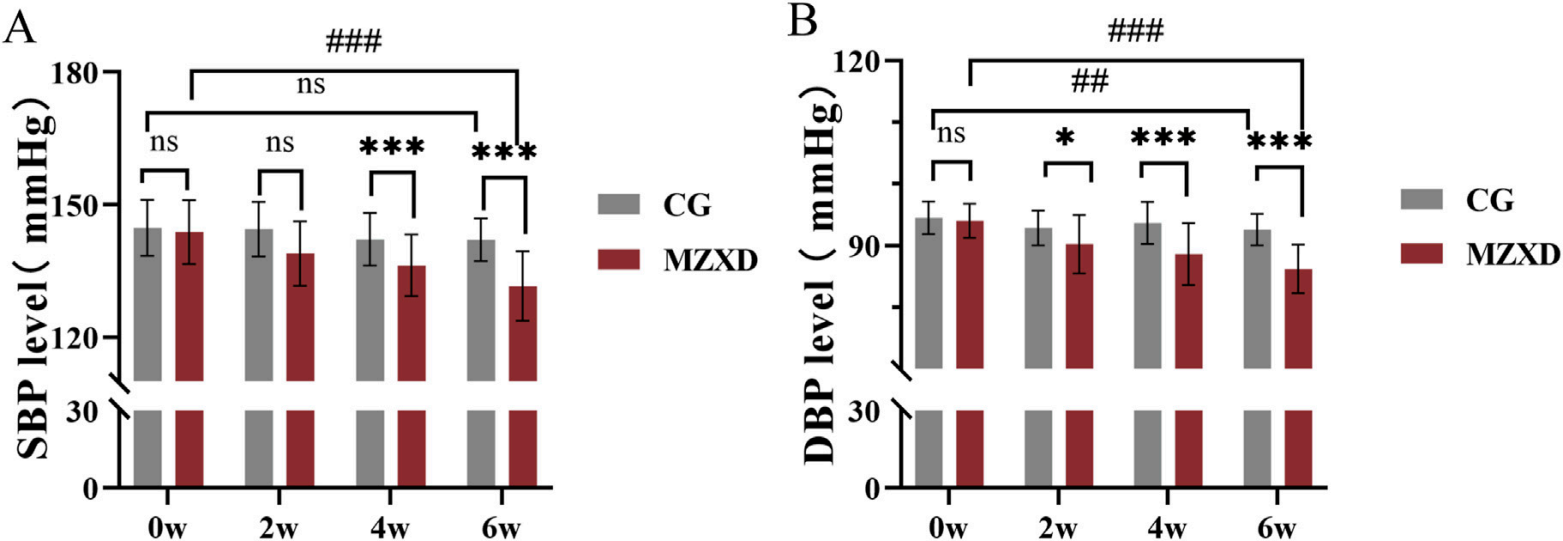
Effect of MZXD on blood pressure in patients with phlegm-dampness grade I hypertension. **(A)** SBP level. **(B)** DBP level. Compared with the control group, *p < 0.05, *p < 0.01; compared with Modified Zexie Decoction before treatment, ##*p* < 0.01; ###*p* < 0.001. CG: control group; MZXD: Treatment group with Modified Zexie Decoction.

To evaluate the antihypertensive effects of MZXD on patients with phlegm-dampness type Stage I hypertension, blood pressure measurements were taken at weeks 0, 2, 4, and 6 in both the CG and MZXD treatment groups. As shown in Figure 1, there were significant changes in systolic blood pressure (SBP) and diastolic blood pressure (DBP) over the course of the measurement periods in both groups. After 6 weeks of healthy lifestyle intervention, the CG showed no significant change in SBP levels (*p* > 0.05, Figure 1A), while DBP levels significantly decreased (*p* < 0.01, Figure 1B). In the MZXD treatment group, after 6 weeks of combined intervention with MZXD and a healthy lifestyle, both SBP and DBP levels were significantly reduced (*p* < 0.001, Figure 1). Furthermore, at weeks 4 and 6, compared to the CG, the MZXD treatment group showed a significantly greater reduction in SBP and DBP levels (*p* < 0.001, Figure 1). These results indicate that while a healthy lifestyle intervention alone has some effect on lowering blood pressure, the addition of MZXD provides more substantial benefits in the treatment of hypertension.

### 3.3 Effects of MZXD on gut microbiota in patients with phlegm-dampness type stage I hypertension

**FIGURE 2:**
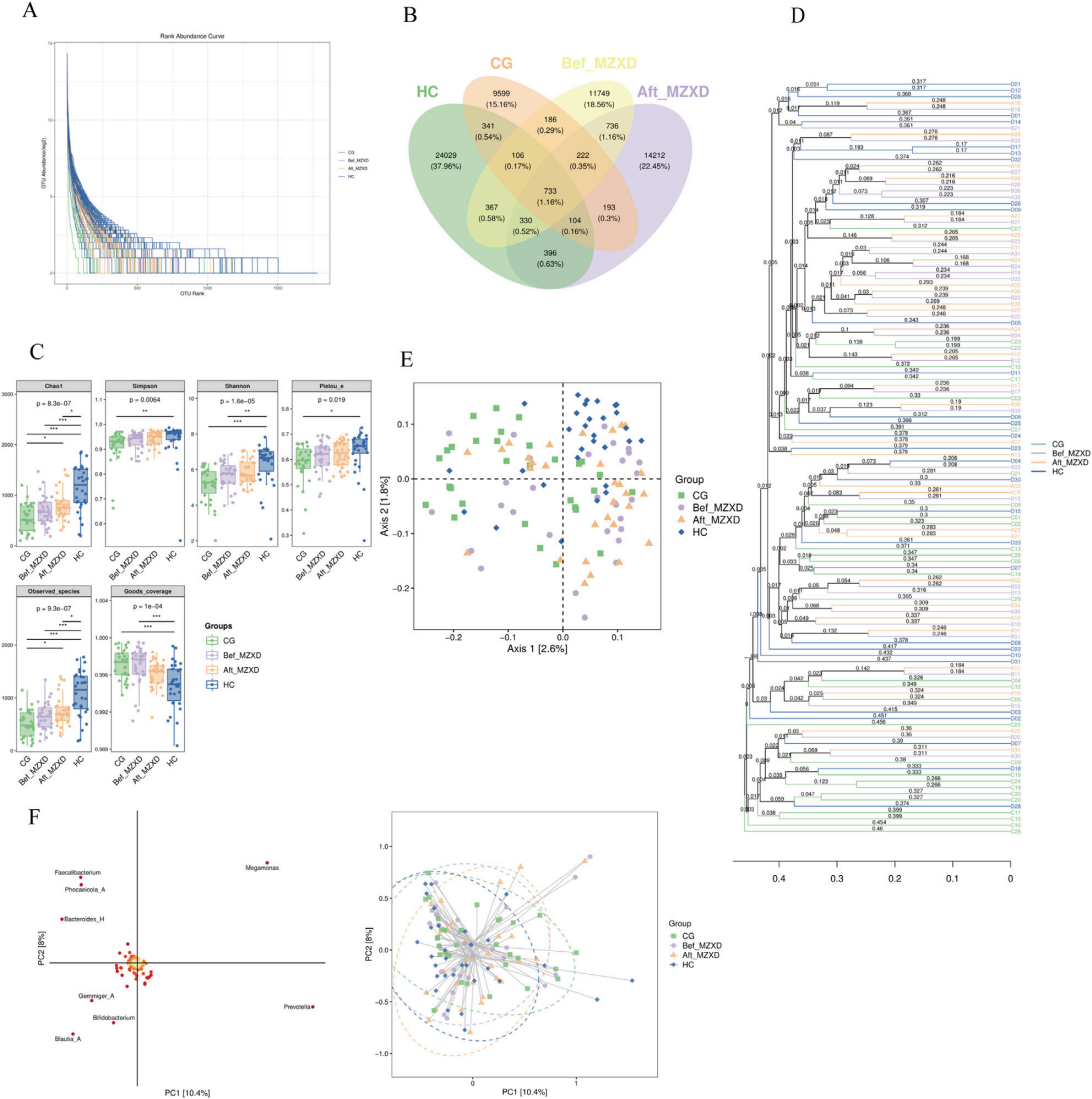
Gut microbiota of subjects in each group. **(A)** Rank-Abundance curve. **(B)** the Wayne diagram of each group ASV/OTU **(C)** the grouped box diagram of Alpha diversity index **(D)** by calculating the distance matrix, all the samples are clustered by hierarchical clustering to form a whole tree diagram indicating the similarity between samples. **(E)** two-dimensional sample sequencing diagram of PCoA analysis **(F)** species load map and sample sequencing diagram of OPLS-DA discriminant analysis. In the intra-group comparison, *p < 0.05, **p < 0.01, ***p < 0.001. CG: control group; Bef_MZXD: group before treatment with Modified Zexie Decoction; Aft_MZXD: group after treatment with Modified Zexie Decoction; HC: healthy control group.

As shown in Figure 2A, the samples from the four groups displayed a wide distribution along the horizontal axis of the Rank-Abundance curve with a gentle downward trend. The curve widths were ranked as HC > Aft_MZXD > Bef_MZXD > CG, indicating uneven species abundance distribution in CG and Bef_MZXD, where a few species dominated while others were less abundant. In contrast, HC and Aft_MZXD showed a more even species distribution, with higher diversity and a greater presence of medium to low-abundance species. Overall, the sequencing data in this experiment were adequate, with evenly distributed and highly abundant species, allowing for further bioinformatics analysis.

As shown in Figure 2B, the number of Operational Taxonomic Units (OTUs) varied significantly among the groups. A total of 60,259 OTUs were identified across all participants, with 11,629 OTUs in the CG, 26,638 OTUs in the HC, 14,629 OTUs in the Bef_MZXD, and 16,299 OTUs in the Aft_MZXD. There were 733 shared species across the four groups, with 9,599 unique species in CG, 24,029 unique species in HC, 11,749 unique species in Bef_MZXD, and 14,212 unique species in Aft_MZXD. Analysis of OTU distribution revealed that HC had the highest number of unique species, reflecting the greatest species richness, whereas CG had relatively fewer unique species, indicating lower species richness. After MZXD treatment, the richness of gut microbiota species in patients with phlegm-dampness type Stage I hypertension increased, suggesting that MZXD has a beneficial regulatory effect on gut microbiota abundance in these patients. These results further support the positive impact of MZXD on the gut microbiota structure, making it more similar to that of the HC.

Alpha diversity indices, including Chao1, Observed species, Shannon, Simpson, Faith’s PD, Pielou’s evenness, and Good’s coverage, are key indicators used to evaluate species richness and diversity. Chao1 and Observed species indices reflect species richness, while Shannon, Simpson, Faith’s PD, and Pielou’s evenness indices reflect species diversity and evenness. The Good’s coverage index indicates the extent of coverage. As shown in Figure 2C, the indices Chao1, Observed species, Shannon, Simpson, Faith’s PD, Pielou’s evenness, and Good’s coverage were significantly higher in HC compared to CG (*p* < 0.05). Although there was no significant improvement in these indices in Aft_MZXD compared to Bef_MZXD (*p* > 0.05), there was an increasing trend in Chao1, Observed species, Shannon, Simpson, and Pielou’s evenness indices, indicating that the diversity and evenness of the microbiota in the MZXD-treated group resembled those of the HC. MZXD treatment can enhance the alpha diversity of the microbiota.

Additionally, beta diversity analyses were conducted, including hierarchical clustering analysis (Figure 2D), Principal Coordinates Analysis (PCoA) (Figure 2E), and Partial Least Squares Discriminant Analysis (PLS-DA) (Figure 2F). Hierarchical clustering analysis showed that Bef_MZXD (orange lines) had a more dispersed distribution, suggesting considerable inter-individual variation in microbial communities before MZXD treatment, while Aft_MZXD (purple lines) showed a certain degree of clustering, indicating that microbial community composition became more similar after MZXD treatment. PCoA results indicated that the species distribution in HC was more concentrated, whereas the species distribution in the other groups was more dispersed, suggesting that HC had distinct species composition, whereas the other groups had more complex species distributions. PLS-DA results indicated no clear separation in the PCA space before and after MZXD treatment, suggesting that MZXD may have had some effect on the microbial community structure.

**FIGURE 3:**
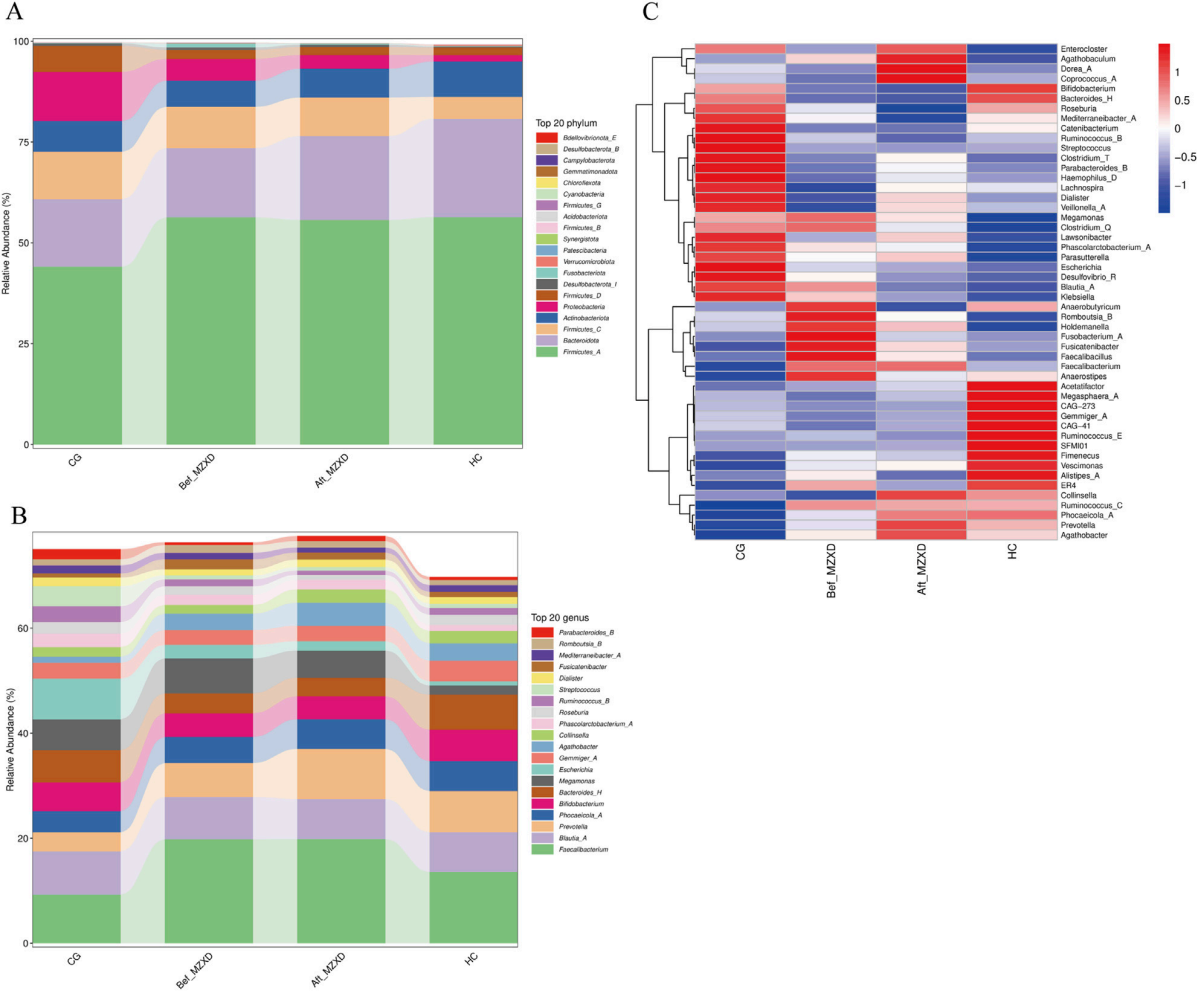
Gut microbiota of subjects in each group. **(A)** abundance composition of gut microbiota at the gate level. **(B)** the abundance composition of intestinal microflora at the genus level. **(C)** the heat map of the relative abundance of bacteria in each group. CG: control group; Bef_MZXD: group before treatment with Modified Zexie Decoction; Aft_MZXD: group after treatment with Modified Zexie Decoction; HC: healthy control group.

To further identify changes in the gut microbiota composition, we compared the bacterial composition at the phylum and genus levels among the four groups. At the phylum level, gut microbiota analysis indicated (Figure 3A) that the intestinal microbiota of the three groups primarily consisted of Firmicutes_A, Bacteroidota, Firmicutes_C, Actinobacteriota, Proteobacteria, and Firmicutes_D. Compared to the CG, the HC showed a significantly higher relative abundance of Firmicutes_A, Bacteroidota, and Actinobacteriota, while the relative abundance of Firmicutes_C and Proteobacteria was significantly lower. Compared to the Bef_MZXD, patients in the Aft_MZXD had an increased relative abundance of Bacteroidota and Actinobacteriota and a significantly reduced relative abundance of Proteobacteria.

At the genus level, the analysis revealed (Figure 3B) that the predominant genera in the gut of the three groups included Faecalibacterium, Blautia_A, and Bifidobacterium, which are typically considered beneficial bacteria. Compared to the CG, the HC showed a significantly higher relative abundance of Faecalibacterium and Prevotella and a significantly lower relative abundance of *Escherichia*. Compared to the Bef_MZXD, the Aft_MZXD had a similar relative abundance of Faecalibacterium, Blautia_A, and Bifidobacterium, with overall consistency in the genus distribution, indicating a high similarity in the microbial community structure between these groups. After MZXD treatment, the relative abundance of Prevotella increased significantly, while the relative abundance of *Escherichia* decreased significantly in the patient’s gut. Furthermore, Figure 3C shows corresponding changes, with a significant reduction in the abundance of Enterocloster, Agathobaculum, and Dorea_A after treatment, whereas other genera, such as Ruminococcus_B and *Streptococcus*, increased in abundance post-treatment, suggesting that MZXD selectively influences different bacterial genera.

### 3.4 Effects of MZXD on SCFAs in patients with phlegm-dampness type stage I hypertension

**FIGURE 4:**
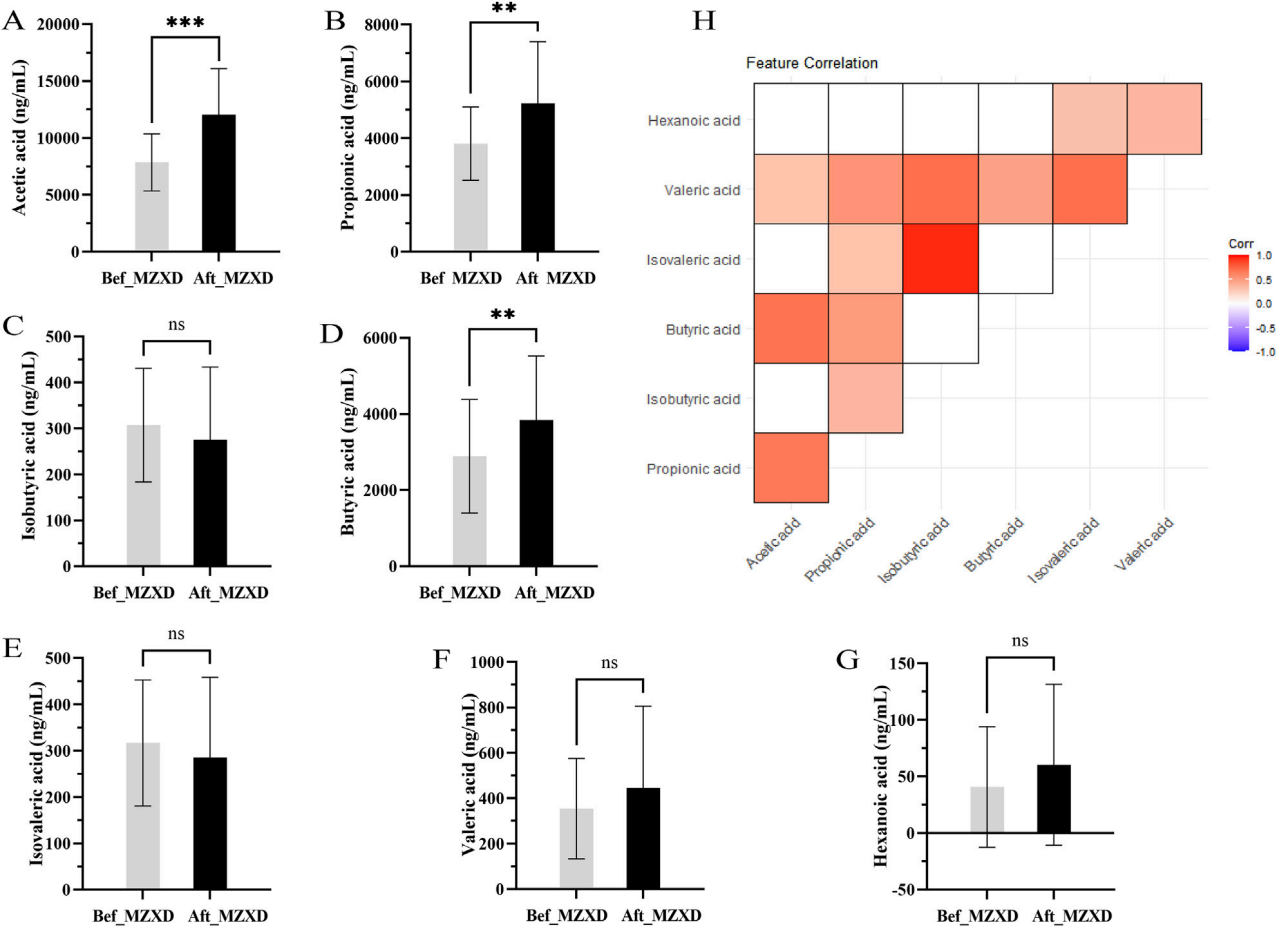
Effect of MZXD on the concentration of SCFAs in phlegm-damp grade I hypertension. **(A)** acetic acid concentration. **(B)** Propionic acid concentration. **(C)** Isobutyric acid concentration. **(D)** butyric acid concentration. **(E)** the concentration of isovaleric acid. **(F)** concentration of pentanoic acid. **(G)** caproic acid concentration. Compared with Modified Zexie Decoction before treatment, **p < 0.01, ***p < 0.001. Bef_MZXD: group before treatment with Modified Zexie Decoction, Aft_MZXD: group after treatment with Modified Zexie Decoction. **(H)** Correlation analysis of various short-chain fatty acids.

Acetate, propionate, and butyrate are the main SCFAs produced in the gut. To evaluate the potential effects of MZXD on the gut microenvironment of patients with phlegm-dampness type Stage I hypertension, we measured the levels of these SCFAs. As shown in Figures 4A–G, the levels of acetate, propionate, and butyrate in the Aft_MZXD were significantly higher compared to the Bef_MZXD (*p* < 0.001, 0.01, 0.01, respectively), while the levels of isobutyrate, isovalerate, valerate, and caproate did not show significant changes. Additionally, correlation analysis among SCFAs metabolites revealed a strong positive correlation between isobutyrate and isovalerate, as well as between acetate and propionate, suggesting that these pairs might serve as potential targets influenced collectively by the treatment (Figure 4H).

### 3.5 Effects of MZXD on immune indicators in patients with phlegm-dampness type stage I hypertension

As shown in Table 4, levels of neutrophils (NE) and peripheral blood mononuclear cells (PBMC), which are components of innate immunity, were significantly reduced after MZXD treatment (*p* < 0.05). The number of natural killer cells (NK) did not show a statistically significant difference before and after MZXD treatment (*p* > 0.05). In terms of adaptive immunity, levels of B cells, T cells, and CD8^+^ T cells decreased following MZXD treatment, while levels of CD25^+^ and CD4^+^CD25^+^ regulatory T cells increased. The number of CD4^+^ T cells did not show a statistically significant difference before and after MZXD treatment (*p* > 0.05). Additionally, after 42 days of MZXD treatment, levels of complement factors C3, C4, and immunoglobulins IgG, IgM, and IgA in the plasma of patients with phlegm-dampness type Stage I hypertension were significantly reduced (*p* < 0.05). Moreover, interleukin-6(IL-6) levels were significantly decreased (*p* < 0.001), while tumor necrosis factor-α (TNF-α), Interferon-γ (IFN-γ), and interleukin-10 (IL-10) levels showed no statistically significant differences (*p* > 0.05).

**TABLE 4:** Effect of MZXD on immune Indexes of phlegm-dampness Grade I Hypertension.

Variable	Bef_MZXD (n = 30)	Aft_MZXD (n = 30)	t/z	*p-*value	*P* (*FDR*)
PBMC (%)	32.53 ± 7.08	29.74 ± 8.19*	2.45	0.021	0.045
NE (%)	61.35 ± 9.19	57.52 ± 13.59*	−2.571b	0.010	0.029
NK (%)	17.63 ± 6.32	17.27 ± 6.73	0.53	0.598	0.684
T cells (%)	68.91 ± 7.43	66.77 ± 7.57*	−2.09	0.037	0.068
B cells (%)	14.86 ± 5.51	12.58 ± 3**	−2.74	0.006	0.025
CD4^+^/CD3^+^ cells (%)	38.65 ± 7.5	38.15 ± 7.36	0.62	0.542	0.65
CD8^+^/CD3^+^ cells (%)	26.19 ± 8.52	27.87 ± 8.81*	−2.05	0.041	0.07
CD25^+^Treg (%)	16.79 ± 3.25	20.85 ± 3.61***	4.72	<0.001	<0.001
CD4^+^CD25 ^high^ Treg (%)	8.55 ± 0.19	9 ± 1.8**	−2.625	0.009	0.03
C4 (g/L)	0.25 ± 0.07	0.23 ± 0.06*	−2.28	0.023	0.046
C3 (g/L)	1.04 ± 0.14	0.98 ± 0.15*	3.33	0.002	0.01
IgM (g/L)	2.54 ± 0.93	2.42 ± 0.9*	−2.56	0.011	0.03
IgG (g/L)	12.5 ± 2.1	11.82 ± 2.73*	−1.98	0.047	0.072
IgA (g/L)	2.54 ± 0.93	2.42 ± 0.9*	−1.98	0.048	0.072
TNF-α (pg/mL)	3.52 ± 3.1	2.82 ± 1.58	−0.36	0.719	0.783
IL-6 (pg/mL)	9.92 ± 11.68	5.7 ± 5.42***	−3.65	<0.001	<0.001
IFN-γ (pg/mL)	4.36 ± 3.29	4.19 ± 1.19	−1.00	0.318	0.424
IL-10 (pg/mL)	5.84 ± 4.1	6.77 ± 6.03	−0.32	0.750	0.783

Note: Compared with Modified Zexie Decoction before treatment, *p < 0.05, **p < 0.01, ***p < 0.001. Bef_MZXD: group before treatment with Modified Zexie Decoction; Aft_MZXD: group after treatment with Modified Zexie Decoction. *p* (FDR) is the Benjamini–Hochberg method for FDR, correction *p* value, which can be used as a reference.

### 3.6 Effects of MZXD on renal function in patients with phlegm-dampness type stage I hypertension

**FIGURE 5:**
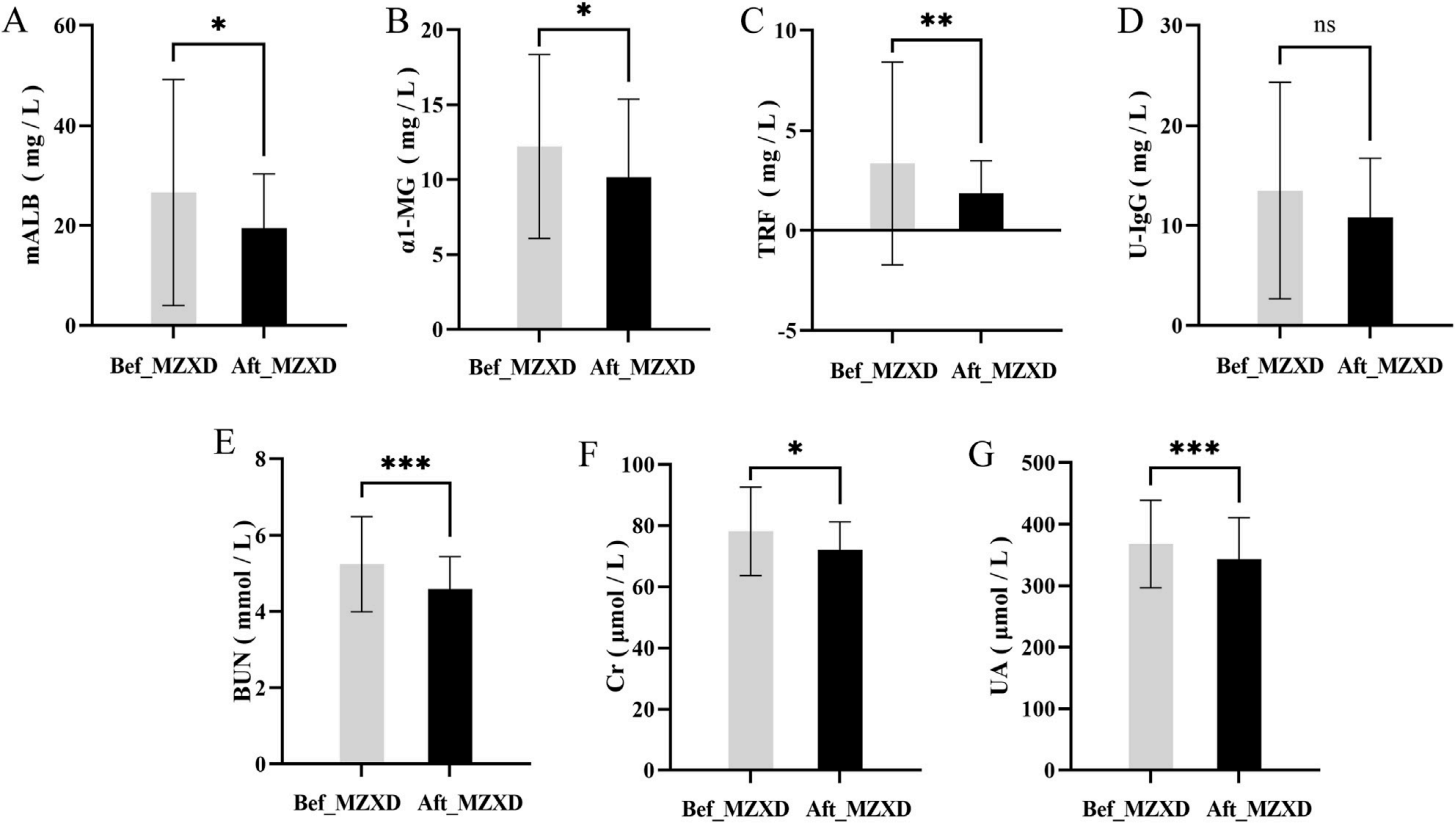
Effect of MZXD on renal function of phlegm-dampness grade I hypertension. **(A)** Microalbumin level. **(B)** α-microglobulin level. **(C)** transferrin level. **(D)** urinary immunoglobulin G level. **(E)** Urea level. **(F)** creatinine level. **(G)** uric acid level. Compared with Modified Zexie Decoction before treatment, *p < 0.05, **p < 0.01, ***p < 0.001. Bef_MZXD: group before treatment with Modified Zexie Decoction, Aft_MZXD: group after treatment with Modified Zexie Decoction.

After MZXD treatment, the renal function indicators in patients with phlegm-dampness type Stage I hypertension showed significant improvement compared to before treatment. Levels of uric acid (UA), blood urea nitrogen (BUN), and creatinine (Cr) were significantly reduced (*p* < 0.001, 0.001, and 0.05, respectively). Interestingly, levels of α1-microglobulin (α1-MG), urinary microalbumin (U-mALB), and urinary transferrin (urinary transferrin) also decreased significantly after treatment (*p* < 0.05). However, there was no statistically significant difference in urinary immunoglobulin G (urinary immunoglobulin G) levels before and after treatment (*p* > 0.05), as shown in Figure 5.

## 4 Discussion

The composition, metabolic functions, and classification characteristics of the gut microbiota in the pre-hypertensive stage closely resemble those observed during hypertension (Li et al., 2017). Phlegm-dampness type Stage I hypertension is closely related to the gut microbiota and its metabolic products. When the local mucosal immunity of the gut is influenced by the intestinal environment, it leads to an imbalance in gut homeostasis. Mature immune cells can be activated by the gut microbiota in surrounding lymphoid organs, triggering a systemic immune response, which plays a significant role in the pathogenesis of hypertension. New antigen presentation by innate immune cells can cause kidney damage, inflammation, and elevated blood pressure. Adaptive immune cells exhibit a pro-inflammatory shift in hypertension, leading to enhanced cytotoxicity and the production of inflammatory cytokines, resulting in impaired vascular function and renal natriuresis (Bomfim et al., 2017; Guzik et al., 2024). In summary, activated immune cells damage the kidneys and vascular endothelium, leading to increased blood pressure.

In this study, we found that MZXD not only exhibited antihypertensive effects in patients with phlegm-dampness type Stage I hypertension but also systematically demonstrated that MZXD could improve the gut microbiota and its metabolic products, inhibit immune cell activation, and ameliorate kidney damage and proteinuria, consistent with previous reports.

MZXD is developed from the classical formula ZXD, which is clinically used to treat dizziness caused by phlegm retention (phlegm-dampness type hypertension), and the herbs in this formula are known to improve gut microbiota (Wang et al., 2019; Zhang et al., 2022). Previous studies have shown that hypertension is associated with gut dysbiosis and a reduced relative abundance of SCFAs-producing bacteria (Luo et al., 2023). Supplementing SCFAs production, either directly or indirectly, can have antihypertensive effects (Ganesh et al., 2018). In a Phase II clinical trial, untreated hypertensive patients who took oral acetate and butyrate showed a clinically relevant reduction in 24-h systolic blood pressure (Jama et al., 2023). The gut microbiota, categorized at the phylum level, primarily consists of Firmicutes, Bacteroidota, Actinobacteriota, and Proteobacteria. A selective decrease in beneficial microbes, such as Firmicutes and Bacteroidota, along with an increase in harmful microbes, including Proteobacteria and Enterobacteriaceae, may exacerbate the progression of cardiovascular diseases (Zhang et al., 2020). Additionally, pro-inflammatory gut microbiota, such as Proteobacteria, can damage the intestinal barrier, leading to immune dysregulation and intestinal inflammation (Chen et al., 2024), which in turn triggers immune responses.

In this study, we found that patients who took MZXD and experienced significant relief of hypertension symptoms had increased abundances of Bacteroidota and Actinobacteriota, and a significantly decreased abundance of Proteobacteria. The relative abundance of *Escherichia* within the Enterobacteriaceae family was also notably reduced. These results indicate that the gut microbiota profile of patients taking MZXD differs from that of untreated hypertensive patients. In phlegm-dampness type Stage I hypertension patients treated with MZXD, the abundance of beneficial bacteria was upregulated, and the production of key SCFAs, such as acetate, propionate, and butyrate, significantly increased, with the gut microbiota composition shifting towards that of healthy individuals.

Regulation of immune responses can reduce elevated blood pressure and prevent hypertensive end-organ damage (Wenzel et al., 2021). Cells of the innate immune system primarily promote blood pressure elevation by affecting renal and vascular function. The key components of adaptive immunity are T and B lymphocytes. Hypertension-specific antigens can be phagocytosed and presented by dendritic cells (DCs) to B cells and T cells, thereby promoting the differentiation of plasma cells and effector T cell subsets (CD8^+^ T cells, CD4^+^ T cells, and regulatory T cells). Abnormal activation of T cells can lead to elevated blood pressure. Studies have shown that diet can directly affect gut microbiota in Dahl salt-sensitive rats, thereby promoting the development of hypertension and renal damage (Abais-Battad et al., 2021). These studies suggest that the immune system serves as a link between gut microbiota and the kidneys, with T cell-mediated adaptive immune mechanisms amplifying salt-sensitive hypertension and renal injury (De Miguel et al., 2010; Mattson et al., 2022). Additionally, immunosuppressants have been found to have therapeutic effects in other animal models of hypertension (Sereti et al., 2020).

In our study, a series of immune indicators showed that after MZXD treatment, levels of PBMCs, neutrophils (NE), T cells, and CD8^+^ T cells were significantly reduced, while levels of CD25^+^ and CD4^+^CD25^+^ regulatory T cells (Tregs) were significantly increased. It is known that Tregs can suppress the function of effector T cells and secrete IL-10 to inhibit inflammatory responses, which is consistent with previous studies. Additionally, we found that levels of B cells, IgG, and IL-6 significantly decreased after MZXD treatment, although IL-10 levels did not show a significant change. Interestingly, activated B cells promote the progression of hypertension, and the IgG they secrete accumulates in the aorta. Elevated serum immunoglobulins are biomarkers of immune activation in hypertension (Chen et al., 2021) and exacerbate vascular damage by promoting inflammatory responses through the modulation of macrophage activation. Furthermore, B cells can contribute to elevated blood pressure and renal damage by regulating water and sodium balance.

In summary, we hypothesize that MZXD may play an important role in the treatment of phlegm-dampness type Stage I hypertension by regulating immune responses, mediating T cell differentiation bias, and inhibiting B cell activation, thereby reducing IgG secretion, suppressing immune activation, and mitigating inflammatory damage.

In our preliminary examinations of patients with phlegm-dampness type Stage I hypertension, we found that they all exhibited some degree of renal dysfunction and proteinuria abnormalities. The kidneys have a unique relationship with blood pressure: renal insufficiency can lead to elevated blood pressure, and elevated blood pressure can accelerate the loss of renal function (Adamczak et al., 2002). Basic experimental and clinical transplantation studies have demonstrated that “blood pressure follows the kidney.” For example, transplanting the damaged kidneys of Dahl salt-sensitive rats into high-salt control rats causes an increase in blood pressure (Oguchi et al., 2014). Similarly, transplanting the kidneys from hypertensive rats into normotensive rats with bilateral nephrectomy results in elevated blood pressure. In patients with primary hypertension and advanced nephrosclerosis who underwent bilateral nephrectomy and received kidney transplants from normotensive donors, hypertension normalized (Rettig et al., 1991a; Rettig et al., 1991b). Therefore, we hypothesize that kidney damage is a key factor in the development of phlegm-dampness type Stage I hypertension.

BUN, UA, and Cr are conventional markers of renal function, and urinary protein excretion is an important marker of cardiovascular risk (Chelliah et al., 2002). In this study, we found that after MZXD treatment, levels of BUN, UA, Cr, and urinary protein significantly decreased in most patients, suggesting an improvement in renal function. This enhancement of renal sodium and water reabsorption could contribute to lowering blood pressure. Conversely, impaired urinary sodium excretion may further polarize T lymphocytes and macrophages towards a pro-inflammatory state, creating a pathogenic feed-forward loop of immune activation and elevated blood pressure (Wen and Crowley, 2019).

Although our clinical data suggest that the gut microbiota and its metabolites in hypertensive patients are associated with inflammation and damage to related end organs, IL-6 and IgG may be key factors. MZXD appears to have a therapeutic effect on phlegm-dampness type Stage I hypertension by targeting the gut-immune-kidney axis. However, the causal relationship is complex.

**FIGURE 6:**
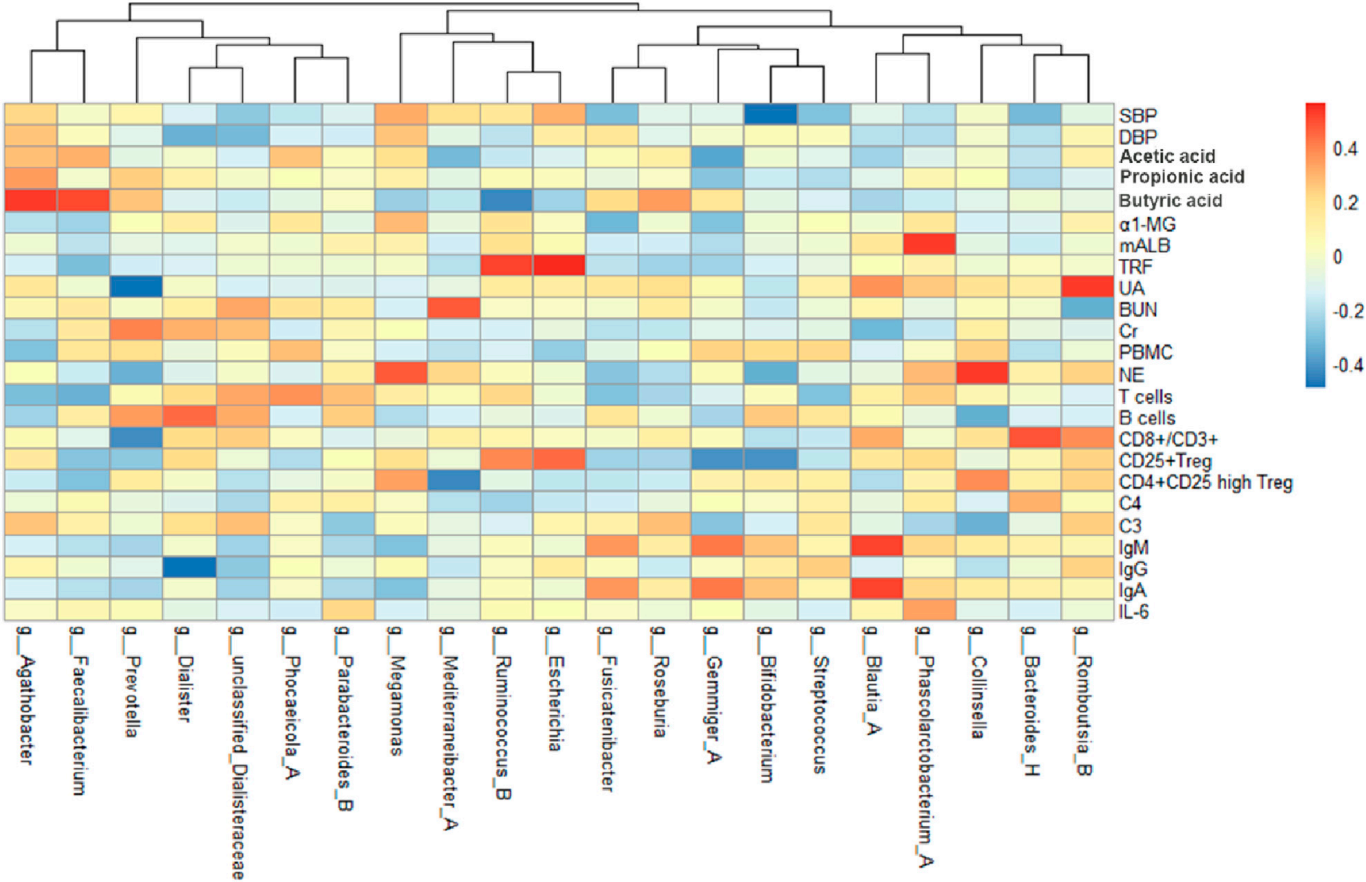
Heat map of correlation analysis before treatment of Modified Zexie Decoction.

By analyzing the correlation between gut microbiota (top 20 bacteria) and blood pressure, immunological indexes, and kidney function, we can observe the changes of different microbiota before and after treatment (Figure 6). After treatment in the MZXD group, the relative abundance of Prevotella and Agathobacter in the intestinal tract was significantly increased, and the relative abundance of *Escherichia* and Megamonas was significantly decreased. The results of correlation analysis showed that the negative correlation between Prevotella and UA was significantly enhanced after treatment, suggesting that Prevotella may play an important role in UA metabolism, and MZXD may help reduce UA levels and improve kidney function by regulating Prevotella abundance. Agathobacter maintained a significant positive correlation with blood pressure before and after treatment. This suggests that the genus may play an important role in the regulation of blood pressure, and the treatment of MZXD may further regulate blood pressure by promoting the increase of its abundance. The positive correlation between *Escherichia* and TRF was significantly enhanced after treatment, suggesting that the genus may be related to the regulation of renal function. In addition, the positive correlation between Megamonas and NE was significantly enhanced after treatment, suggesting that Megamonas may be involved in the regulation of the immune system. In conclusion, after MZXD treatment, there was a strong correlation between the significantly changed bacterial genera and immune indexes and renal function markers, suggesting that MZXD treatment strengthened the role of the “gut-immune-kidney axis”.

Our data can only demonstrate correlation; the precise mechanisms remain to be validated by basic experiments. Limitations of this study include the use of a lifestyle intervention only and no placebo control for CG, which may introduce placebo effect bias due to participants’ psychological expectations. At the same time, due to ethical considerations, CG did not perform blood biochemical testing, and there was a lack of comprehensive physiological evaluation of these subjects. Although the above design may weaken the rigor of the interpretation of the results, we ensure the reliability of the core conclusions by implementing rigorous longitudinal physiological monitoring (including multi-dimensional analysis of blood pressure, microbiome, SCFA, and blood immunity indicators) in the MZXD treatment group. Future studies recommend the use of double-blind, placebo-controlled, and parallel-controlled classical formulas, and the specific efficacy of MZXD through randomized clinical trials with larger sample sizes.

In addition, dietary and lifestyle differences also affect the changes in intestinal microbiota and its metabolites, and this study observed that MZXD has a significant effect on the improvement of intestinal microbiota, immune regulation, and renal function in patients with phlegm-damp grade I hypertension, but the influence of confounding factors should be carefully considered. Although we have maximized patients’ medication compliance and lifestyle habits through regular telephone follow-up visits and healthy lifestyle guidance, subtle differences in dietary structure and potential non-adherence behaviors between individuals may still have an impact on gut microbiota composition and metabolites. Due to the objective adherence monitoring methods used in this study, these factors may affect the interpretation of the results as unquantified confounding variables. Future studies can further improve the control of confounding factors through standardized dietary interventions, electronic medication monitoring techniques, or drug metabolomics analysis. However, this study is a real study, and despite individual differences, the overall trend in the MZXD treatment group is significantly better than CG.

## 5 Conclusion

Based on the above data, we propose that MZXD improves gut microbiota and increases the levels of key SCFAs through the gut-immune-kidney axis, thereby restoring gut homeostasis, inhibiting immune activation, reducing inflammatory damage, and mitigating renal injury and proteinuria, ultimately exerting antihypertensive effects. MZXD demonstrates good efficacy and unique therapeutic potential. It not only provides a new treatment option for hypertensive patients, particularly those with phlegm-dampness type Stage I hypertension but also serves as an effective adjunct to conventional antihypertensive medications in clinical practice. Its multi-target mechanism of action and favorable safety profile make it a promising traditional Chinese medicine with broad applications, especially for patients who respond poorly to conventional drugs or experience side effects.

Future research should focus on large-scale, multicenter clinical trials to validate the efficacy and safety of MZXD in a broader patient population. Additionally, studies should explore the applicability of MZXD in different types of hypertensive patients and investigate its synergistic effects with conventional treatment modalities. Further mechanistic studies will help elucidate the molecular mechanisms of MZXD, providing a stronger theoretical foundation for its integration into comprehensive hypertension management.

## Data Availability

The data presented in the study are deposited in the NCBI (SRA) repository, accession number PRJNA1274193.

## Abbreviations

BMI, body mass index; BUN, blood urea nitrogen; Cr, creatinine; C4, Complement factor 4; C3, Complement factor 3; DCs, dendritic cells; DBP, diastolic blood pressure; GC-MS, gas chromatography-mass spectrometry; HPLC-MS, high performance liquid chromatography-mass spectrometry; IL-6, Interleukin-6; IFN-γ, Interferon-γ; IL-10, Interleukin-10; IgM, Immunoglobulin M; IgG, Immunoglobulin G; IgA, Immunoglobulin A; MZXD, Modified Zexie Decoction; NE, neutrophils; NK, Natural killer; PBMC, Peripheral blood mononuclear cell; SCFAs, short-chain fatty acids; SBP, systolic blood pressure; TRF, urinary transferrin; TCM, traditional Chinese medicine; TNF-α, Tumor Necrosis Factor-α; U-mALB, urinary microalbumin; UA, uric acid; U-IgG, urinary immunoglobulin G; ZXD, Zexie Decoction; α1-MG, α1-microglobulin.
