# Supplementary material for: Modified Zexie decoction improves phlegm-dampness type stage I hypertension by regulating the gut-immune-kidney axis
Source: Front Pharmacol. 2025 Jun 18;16:1578815. doi: 10.3389/fphar.2025.1578815 (PMC12215698; doi:10.3389/fphar.2025.1578815)
Supplement: Supplementary file 1 [file Supplementaryfile1.docx]

**Modified Zexie Decoction Improves Phlegm-Dampness Type Stage I Hypertension by Regulating the Gut-Immune-Kidney Axis**

Zeqi-Hu ^a^, Jiujie-Jia ^a^, Yiyan-Su ^c^, Yihao-Gu ^c^, Bingbing-Cheng ^a^, Ninghua-Jiang ^b,^*

a College of Pharmaceutical Science, Zhejiang University of Technology, Hangzhou, Zhejiang 310014, China

b Department of Pharmacy, The Second Affiliated Hospital of Jiaxing University, Jiaxing, Zhejiang 314000, China

c College of Pharmaceutical Science, Zhejiang Chinese Medical University, Hangzhou, Zhejiang 310053, China

**Supplemental Data**

**Table of Contents**

[**Table S1 Blood pressure level** 2](#_Toc187850457)

[**Table S2 Short chain fatty acid level** 2](#_Toc187850458)

[**Table S3 Related indexes of renal function** 2](#_Toc187850459)

[**Table S4 Immune related index level** 3](#_Toc187850460)

[**Preparation and composition Identification of MZXD decoction** 3](#_Toc187850461)

**Table S1 Blood pressure level**

| Group | n | SBP | | | | *t* | *p* |
| --- | --- | --- | --- | --- | --- | --- | --- |
|  |  | 0w | 2w | 4w | 6w |  |  |
| CG | 30 | 144.77±6.34 | 142.67±6.2 | 142.17±5.96 | 142.07±4.8 | 1.859 | 0.068 |
| MZXD | 30 | 143.83±7.22 | 139.43±7.09 | 136.27±7 | 131.6±7.9 | 6.259 | <0.001 |
| *t* |  | 0.532 | 1.880 | 3.516 | 3.516 |  |  |
| *p* |  | 0.597 | 0.065 | <0.001 | <0.001 |  |  |

| Group | n | DBP | | | | *t* | *p* |
| --- | --- | --- | --- | --- | --- | --- | --- |
|  |  | 0w | 2w | 4w | 6w |  |  |
| CG | 30 | 94.5±2.65 | 92.87±2.8 | 93.67±3.41 | 92.6±2.54 | 2.835 | 0.006 |
| MZXD | 30 | 94.03±2.76 | 90.23±4.73 | 88.63±5.03 | 86.2±3.95 | 9.248 | <0.001 |
| *t* |  | 0.668 | 2.623 | 4.539 | 7.462 |  |  |
| *p* |  | 0.5067 | 0.0111 | <0.001 | <0.001 |  |  |

**Table S2 Short chain fatty acid level**

| Variable | Bef_MZXD (n=30) | Aft_MZXD (n=30) | t/z | *p-*value |
| --- | --- | --- | --- | --- |
| Acetic acid | 7867.45±2510.34 | 12045.26±4047.48 | -4.49 | ＜0.001 |
| Propionic acid | 3808.33±1290.94 | 5233.64±2167.39 | -3.07 | 0.005 |
| Isobutyric acid | 307.22±123.53 | 275.96±157.83 | 1.07 | 0.295 |
| Butyric acid | 2891.09±1490.68 | 3841.94±1686.28 | -2.98 | 0.006 |
| Uric acid | 367.92±71.23 | 343.14±67.85 | -3.312b | ＜0.001 |
| Isovaleric acid | 316.86±135.86 | 285.38±172.81 | 1.03 | 0.313 |
| Pentanoic acid | 354.51±221.37 | 446.11±359.52 | -1.76 | 0.089 |
| Caproic acid | 40.78±53.31 | 60.47±71.15 | -1.92 | 0.054 |

**Table S3 Related indexes of renal function**

| Variable | Bef_MZXD (n=30) | Aft_MZXD (n=30) | t/z | *p-*value |
| --- | --- | --- | --- | --- |
| α 1-microglobulin | 12.23±6.14 | 10.17±5.23 | 2.56 | 0.016 |
| Urinary microprotein | 26.61±22.62 | 19.5±10.84 | -2.37 | 0.018 |
| Urinary transferrin | 3.36±5.07 | 1.87±1.62 | -2.76 | 0.006 |
| Urinary immune protein G | 13.49±10.82 | 10.83±5.9 | -1.82 | 0.069 |
| Uric acid | 367.92±71.23 | 343.14±67.85 | -3.312b | ＜0.001 |
| Urea | 5.24±1.25 | 4.59±0.85 | 4.65 | ＜0.001 |
| Creatinine | 78.15±14.5 | 72.17±9.09 | -2.478b | 0.013 |

**Table S4 Immune related index level**

| Variable | Bef_MZXD (n=30) | Aft_MZXD (n=30) | t/z | *p-*value |
| --- | --- | --- | --- | --- |
| PBMC (%) | 32.53±7.08 | 29.74±8.19* | 2.45 | 0.021 |
| NE (%) | 61.35±9.19 | 57.52±13.59* | -2.571b | 0.010 |
| NK (%) | 17.63±6.32 | 17.27±6.73 | 0.53 | 0.598 |
| T cells (%) | 68.91±7.43 | 66.77±7.57* | -2.09 | 0.037 |
| B cells (%) | 14.86±5.51 | 12.58±3** | -2.74 | 0.006 |
| CD4^+^/CD3^+^ cells (%) | 38.65±7.5 | 38.15±7.36 | 0.62 | 0.542 |
| CD8^+^/CD3^+^ cells (%) | 26.19±8.52 | 27.87±8.81* | -2.05 | 0.041 |
| CD25^+^Treg (%) | 16.79±3.25 | 20.85±3.61*** | 4.72 | ＜0.001 |
| CD4^+^CD25 ^high^ Treg (%) | 8.55±0.19 | 9±1.8** | -2.625 | 0.009 |
| C4 (g/L) | 0.25±0.07 | 0.23±0.06* | -2.28 | 0.023 |
| C3 (g/L) | 1.04±0.14 | 0.98±0.15* | 3.33 | 0.002 |
| IgM (g/L) | 2.54±0.93 | 2.42±0.9* | -2.56 | 0.011 |
| IgG (g/L) | 12.5±2.1 | 11.82±2.73* | -1.98 | 0.047 |
| IgA (g/L) | 2.54±0.93 | 2.42±0.9* | -1.98 | 0.048 |
| TNF-α (pg/mL) | 3.52±3.1 | 2.82±1.58 | -0.36 | 0.719 |
| IL-6 (pg/mL) | 9.92±11.68 | 5.7±5.42*** | -3.65 | ＜0.001 |
| IFN-γ (pg/mL) | 4.36±3.29 | 4.19±1.19 | -1.00 | 0.318 |
| IL-10 (pg/mL) | 5.84±4.1 | 6.77±6.03 | -0.32 | 0.750 |

**Preparation and composition Identification of MZXD decoction**

MZXD is composed of three kinds of traditional Chinese medicine, including Zexie, Baizhu and Shanzha. All herbs are purchased from the Pharmacy Department of the second affiliated Hospital of Jiaxing University. The MZXD decoction was prepared according to the following process: the plant medicine was gathered together, soaked in 2 L distilled water for half an hour, boiled over high heat, and continued to boil for 1 hour; after filtering the liquid, the drug residue was added to distilled water and boiled again for 40 min; to mix the two kinds of filtrate, evaporated, diluted with methanol of 1 g to 10 mL, sterilized, filtered with a sterile filter with a pore diameter of 0.22 μ m, and then stored at-80 °C.

In order to clarify the material basis of the decoction, the components of MZXD were comprehensively identified by ultra high performance liquid chromatography-tandem mass spectrometry (UPLC-MS/MS). The identified components were shown in the **excel file named Supplemental Data 2.**
